# A role for *p16*^High^ immune cells in disease tolerance as a crucial defence strategy

**DOI:** 10.1098/rstb.2025.0323

**Published:** 2026-11-12

**Authors:** Francisco Triana-Martinez, Dmitry V. Bulavin

**Affiliations:** IRCAN, Université Côte d'Azur, INSERM or CNRS, Nice, Provence-Alpes-Côte d'Azur, France

**Keywords:** *p16*, beneficial senescence, disease tolerance, ageing, healthspan

## Abstract

In the context of infectious diseases, it is well established that older individuals are more susceptible to pathogens. However, an organism's survival does not rely solely on its ability to eliminate pathogens, a process collectively referred to as resistance. An additional protective strategy, known as disease tolerance, has evolved in multiple species. In response to severe inflammation and tissue damage, disease tolerance allows the host to limit the negative impact of infection without directly reducing pathogen burden. It relies on mechanisms that control tissue damage and stress response to preserve host health and physiological fitness. In recent years, the concept of disease tolerance has gained attention, as it offers a complementary approach to traditional resistance-based strategies in managing inflammation, tissue damage and infection, ultimately improving survival outcomes. However, the full spectrum of disease tolerance mechanisms and the therapeutic opportunities to enhance them remain incompletely understood. In this context, we will discuss recent insights into disease tolerance and propose a role for immune cells exhibiting senescence-like features—specifically, the activation of the *p16* gene—in the positive regulation of disease tolerance, with implications for treating various pathological conditions and potentially extending healthspan.

This article is part of the theme issue ‘Developmental circuits in ageing’.

## 1. Introduction

Ageing is a multifactorial biological process characterized by declining physiological function, increased susceptibility to disease and a reduced capacity to adapt to environmental stressors, all of which contribute to a higher risk of mortality. In the context of infectious diseases, it is well established that older individuals are more susceptible to pathogens. However, an organism's survival does not rely solely on its ability to eliminate pathogens, a process collectively referred to as resistance. An additional protective strategy, known as disease tolerance, has evolved in multiple species. In response to severe inflammation and tissue damage, disease tolerance allows the host to limit the negative impact of infection without directly reducing pathogen burden. It relies on mechanisms that control tissue damage and stress response to preserve host health and physiological fitness. In recent years, the concept of disease tolerance has gained attention because it offers a complementary approach to traditional resistance-based strategies in managing inflammation, tissue damage and infection, ultimately improving survival outcomes. However, the full spectrum of disease tolerance mechanisms and the therapeutic opportunities to enhance them remain incompletely understood. In this context, we will discuss recent insights into disease tolerance and propose a role for immune cells exhibiting senescence-like features—specifically, the activation of the *p16* gene—in the positive regulation of disease tolerance, with implications for treating various pathological conditions and potentially extending healthspan.

Originally described as the ability of plants to withstand loss of grain production following pathogen infection [1], disease tolerance is increasingly recognized as a defence mechanism that enables different species to withstand infections without directly targeting the invading pathogens. Unlike resistance mechanisms, which aim to eliminate infectious agents, disease tolerance focuses on mitigating the harmful effects of pathogens on the host. Ultimately, this form of protection comes down to limiting the extent of tissue and organ damage, as well as promoting tissue remodelling and organ repair mechanisms during the recovery phase.

Adaptive homeostasis, defined as the transient expansion or contraction of the homeostatic range for any given physiological parameter, provides a first-line response to exposure to sub-toxic, non-damaging signalling molecules or events (figure 1). When the exposure is more substantial and results in noticeable tissue damage, the mechanism of disease tolerance may be engaged to restore homeostatic balance. Both adaptive homeostasis and disease tolerance decline with ageing, ultimately reducing the organism's capacity to respond effectively to harmful factors. While disease tolerance has traditionally been viewed in the context of host responses to pathogens such as viruses and bacteria, it is now increasingly recognized as relevant to non-infectious and non-inflammatory conditions as well. It is a highly dynamic and multifactorial process, involving multiple cell types across tissues and regulating diverse molecular pathways to protect the host from functional deterioration. Some aspects of disease tolerance have been identified, including the maintenance of tissue function despite pathogen load, the regulation of the inflammatory response to prevent excessive damage [2,3], the control of tissue repair and regeneration pathways [2,4] and the modulation of metabolism to adapt to infection stress [5,6].

**Figure 1.**
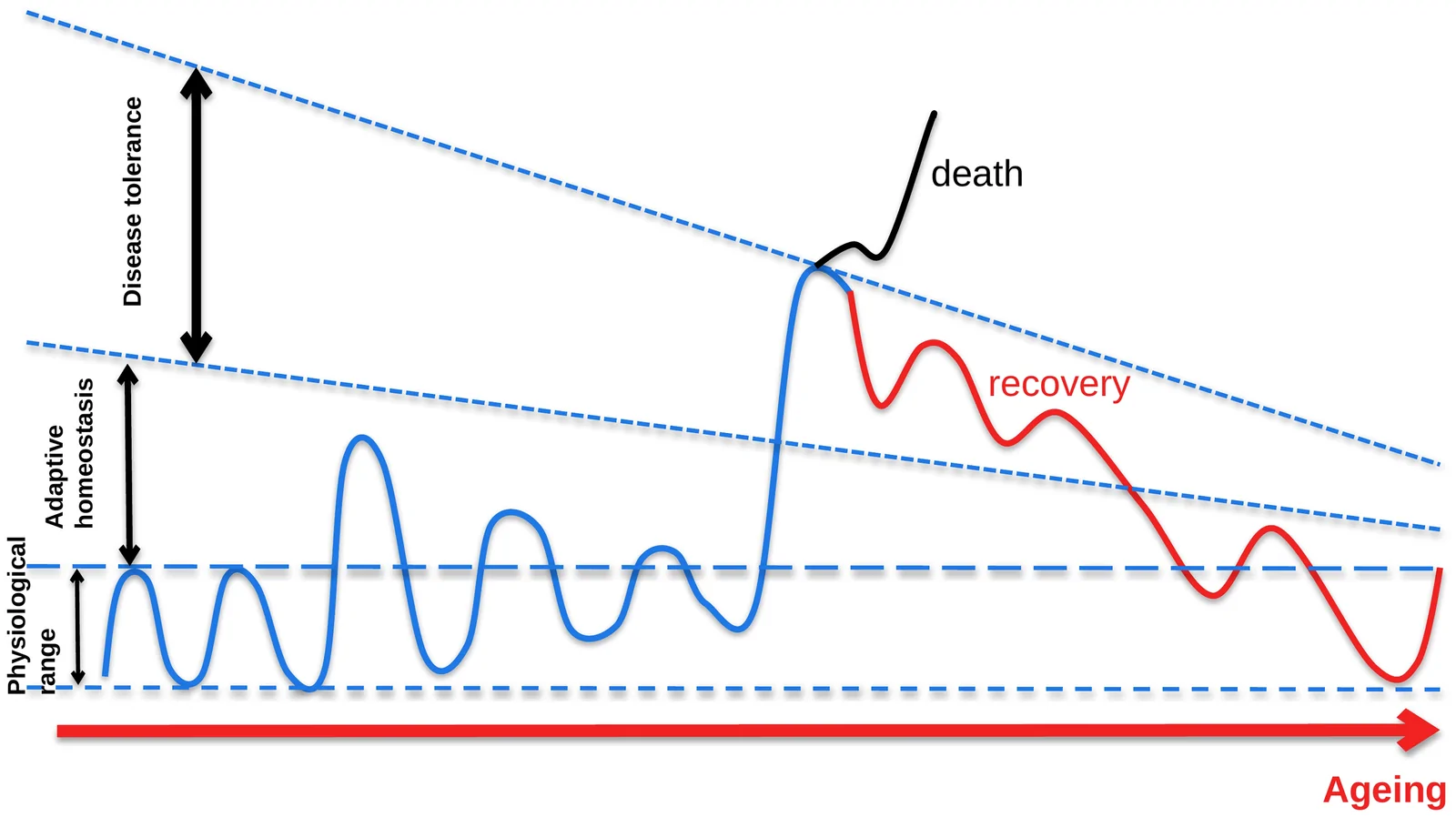
A role for adaptive homeostasis and disease tolerance in healthspan. Organisms normally function within a physiological range. Under certain conditions, organisms can undergo a transient expansion or contraction of this range, developing adaptive homeostasis. This process provides a first-line response and defence to sub-toxic, non-damaging events. When the exposure is more substantial and causes noticeable tissue damage, the mechanism of disease tolerance may be engaged to restore homeostatic balance. Both adaptive homeostasis and disease tolerance decline with ageing, ultimately reducing the organism's capacity to respond effectively to harmful factors.

While we still have a limited view of the full spectrum of mechanisms, it is becoming clear that identifying strategies to enhance disease tolerance could provide an important tool to improve treatment of multiple pathological conditions. The elimination of pathogens through inflammatory mediators, such as cytokines and chemically reactive species like reactive oxygen species (ROS), nitric oxide or hypochlorous acid (produced by neutrophil myeloperoxidase), is an energy-intensive process that can cause additional tissue damage if not resolved in time. One prominent example is hypercytokinaemia, or cytokine storm, which is a major factor contributing to lung damage and intensive care admissions in severe COVID-19 patients. This is especially true in individuals with comorbidities or advanced age, where a state of chronic low-grade inflammation is often observed [7]. As a result, clinicians and researchers are beginning to explore therapies that aim to enhance disease tolerance, particularly in vulnerable populations [8]. A deeper understanding of the cellular and molecular mechanisms underlying disease tolerance is essential. These insights should be incorporated not only into our understanding of infectious diseases but also into the broader definitions of diseases characterized by chronic inflammation, such as autoimmune disorders, cancer, neurodegenerative diseases and, ultimately, ageing itself.

## 2. Stress responses as pivotal resistance and disease tolerance mechanisms

Stress response pathways, well-studied in different contexts, also link to disease tolerance through their activation during pathogen infection. Pattern recognition receptor activation triggers inflammatory resistance mechanisms as the primary response, followed by stress responses that, when balanced, contribute to restoring tissue homeostasis. Oxidative stress and hypoxia responses are two key pathways that control both resistance and tolerance, depending on the tissue, pathogen and context. Notably, some pathogens could also exploit these responses to their advantage.

The oxidative stress response is activated following the induction of resistance molecules like NADPH oxidase, which produces ROS such as superoxide anion and hydrogen peroxide to kill pathogens. Innate immune cells (macrophages and neutrophils) produce ROS as a first defence, and mitochondrial ROS production also increases during infection. ROS amplify immune responses by activating the NLRP3 inflammasome. Epithelial cells produce ROS too, mainly as signals to recruit immune cells rather than for direct pathogen killing [9]. Excessive ROS trigger a protective response involving the transcription factor NRF2, which, when released from its inhibitor KEAP-1, activates antioxidant genes. NRF2 activation in macrophages boosts phagocytosis and tissue repair by reducing inflammatory cytokine expression [10]. Other immune cells, like T cells, also induce NRF2 during chronic infection, though its role is complex; in CD8+ T cells, NRF2 helps to maintain cell fitness but can impair pathogen control and reduce immune checkpoint molecules [11]. This suggests that NRF2 and similar pathways act as molecular switches toggling between resistance and tolerance, with details still under investigation.

Alongside oxidative stress, hypoxia-inducible factor 1 alpha (HIF1α) is a master regulator of hypoxia responses and integrates resistance and tolerance mechanisms. HIF1α is activated by inflammatory cytokines (TNFα and IL1β), Toll-like receptor 4 (TLR4) signalling and low tissue oxygen due to pathogen metabolism. In bacterial infections, HIF1α promotes pro-inflammatory molecules and antimicrobial factors, driving macrophages to an M1 state to better function in hypoxic environments. However, myeloid-specific HIF1α deletion reduces inflammation and protects against lipopolysaccharide (LPS)-induced sepsis, highlighting its double-edged role [12,13]. In epithelial cells, HIF1α activation supports tissue homeostasis by dampening inflammation; in the intestine, it maintains barrier integrity and controls microbiota balance [14]. Loss of HIF leads to gut dysbiosis, promoting gut leakage and endotoxaemia [15]. Some viruses manipulate hypoxia pathways: in lungs, SARS-CoV-2 and H1N1 reduce type II interferon expression through HIF activity, aiding viral replication [16,17].

Overall, stress pathways can serve as disease tolerance mechanisms in specific contexts and to particular pathogen types but are not universal. Their role depends on tissue, cell type and the evolutionary history between host and pathogen, reflecting a complex balance between resistance and disease tolerance.

## 3. DNA damage responses in disease tolerance

Cellular stress responses, such as the DNA damage response (DDR) and unfolded protein response (UPR), are essential for maintaining tissue homeostasis by repairing DNA lesions and clearing misfolded proteins. The DDR detects DNA alterations and initiates downstream pathways involved in DNA repair, senescence or cell death. During infection, inflammation and ROS induce DNA damage, leading to the release of damage-associated molecular patterns (DAMPs) that activate innate immune receptors such as cGAS and TLR9, thereby amplifying inflammatory signalling. Importantly, controlled modulation of the DDR within a physiological range can exert tissue-protective effects, which could be detrimental in another context [18]. For example, treatment with anthracyclines such as epirubicin improves survival in septic mice through activation of ataxia telangiectasia mutated (ATM) kinase, which may promote epigenetic reprogramming and autophagy while suppressing pro-inflammatory mediators including TNF, IL-1β and IL-6 [19]. In turn, mice lacking ATM or activating transcription factor 7 (ATF7) fail to mount this tolerance response, highlighting the importance of DDR pathways [19].

## 4. Protein damage response coupled with resistance and disease tolerance

Proteostasis mechanisms, including protein folding and quality control, extend their roles during infection by promoting disease tolerance through limiting immunopathology without affecting pathogen load [20,21]. Chaperone networks, stress responses and protein degradation pathways coordinate tissue protection by reducing proteotoxic stress, modulating inflammation and preserving organ function. Key proteostatic pathways include the endoplasmic reticulum (ER) UPR, cytosolic heat shock response (HSR) and autophagy [22].

The ER UPR (via inositol-requiring enzyme 1 (IRE1)–X-box binding protein 1 (XBP1), protein kinase R-like endoplasmic reticulum kinase (PERK) and activating transcription factor 6 (ATF6)) shifts function during infection, balancing protein folding with inflammatory regulation. In malaria models, UPR reprogramming of immune cells supports tolerance by suppressing pro-inflammatory cytokines and enhancing phagocytosis [23]. XBP1 splicing increases ER-associated degradation components and modulates NF-κB signalling, fine-tuning inflammation [24]. ATF6 activation during influenza helps fold viral proteins while restraining inflammasome activation, preventing excessive immune cell death [25]. This temporal separation of proteostasis and inflammation allows tissue repair and tolerance.

Mitochondrial UPR (UPRmt) also mitigates infection-induced oxidative stress by upregulating chaperones (HSP60 and HSP10) that maintain electron transport chain function despite ROS [26]. The HSR–UPRmt axis exemplifies how stress chaperones evolved dual roles in protein quality control and metabolic resilience during infection.

Autophagy remodels during infection to clear immunogenic protein aggregates selectively. For example, during *Plasmodium* infection, autophagy receptors p62/SQSTM1 and NBR1 target oxidized haemoglobin derivatives for degradation, preventing inflammasome activation in splenic macrophages [27].

These observations highlight proteostasis as a molecular foundation of disease tolerance, balancing protein quality control with physiological adaptation. ER stress sensors act as inflammation gatekeepers, while autophagy maintains immune homeostasis. Understanding these mechanisms opens therapeutic opportunities to enhance host resilience without promoting pathogen resistance. Future work integrating organ-level proteostasis with single-cell analysis will be key to developing targeted tolerance therapies.

## 5. Immune and non-immune components of disease tolerance

Disease tolerance involves a complex interplay between immune and non-immune cells, shaped largely by the type of pathogen and the host's response. Resistance mechanisms clear pathogens, whereas disease tolerance modulates mechanisms to prevent excessive tissue damage and promote tissue repair.

### (a) Innate lymphoid cells

Innate lymphoid cells (ILCs) are innate immune cells that lack antigen-specific receptors but functionally resemble T helper subsets. They are divided into ILC1, ILC2 and ILC3 based on the cytokines produced. ILC2s maintain epithelial barriers and promote tissue repair by releasing IL-5, IL-13 and amphiregulin, which stimulate mucus production, eosinophil recruitment and epithelial regeneration—vital for limiting inflammation in organs like the lungs and skin. Amphiregulin binds epidermal growth factor receptor (EGFR) to aid tissue recovery and dampen excessive immune responses [28]. ILC3s produce IL-22, supporting epithelial survival and tight junction integrity, especially in the gut and lungs, which enhances barrier function without directly targeting pathogens [29,30]. ILCs interact with epithelial cells, dendritic cells and T cells, integrating early signals at mucosal surfaces to maintain tissue resilience.

### (b) Macrophages

Macrophages are key regulators of disease tolerance through their ability to switch between pro-inflammatory (M1-like) and anti-inflammatory (M2-like) states. Initially, M1 macrophages kill pathogens using reactive species and pro-inflammatory cytokines like TNF-α and IL-6. However, prolonged M1 activation risks tissue damage, so macrophages transition to M2-like phenotypes that secrete IL-10, transforming growth factor-β (TGF-β) and growth factors (e.g. VEGF and IGF-1), which help to resolve inflammation and promote tissue repair [31]. For example, during malaria, macrophages are reprogrammed to protect vascular integrity and minimize tissue damage, a host adaptation that limits severe disease [27]. Macrophages also clear apoptotic cells (efferocytosis), preventing secondary necrosis and further inflammation [32]. Tissue-resident macrophages, which are adapted to their specific organ environments, sense metabolic stress and respond to local signals to maintain homeostasis, balancing pathogen control with tissue protection [33,34].

### (c) Neutrophils

Neutrophils are frontline defenders known for pathogen killing through degranulation, ROS and neutrophil extracellular traps. While their actions can cause tissue damage, neutrophils also contribute to disease tolerance by regulating their activation, producing anti-inflammatory cytokines like IL-10 [35,36] and supporting tissue repair via growth factors, such as VEGF and HGF [37]. Apoptotic neutrophils promote macrophage polarization towards repair phenotypes [38]. Recent studies show that neutrophils can develop ‘trained immunity’ after exposure to stimuli like β-glucan, generating regulatory subsets that protect tissue integrity during infections like influenza [36]. During helminth infections, microbiota drive gut-resident CD8+ T cells to produce interferon-γ (IFN-γ), recruiting neutrophils to limit tissue injury [39]. Neutrophil heterogeneity and plasticity suggest roles in resolving inflammation and maintaining homeostasis beyond their classical destructive reputation.

### (d) Natural killer cells

Natural killer (NK) cells are innate cytotoxic lymphocytes critical for early pathogen control. Beyond killing infected cells, NK cells secrete cytokines like IFN-γ and IL-10, which have context-dependent regulatory effects [40]. IL-10-producing NK subsets help resolve inflammation and protect tissue integrity without affecting pathogen load, as seen in parasitic infections [41]. NK cells also influence dendritic cell maturation and shape T cell responses to avoid immune overactivation. In chronic infections and sepsis, NK cells can become functionally exhausted or adapt to a less cytotoxic but more regulatory state, supporting disease tolerance by preventing immune-mediated tissue injury, especially at mucosal sites vulnerable to inflammation [42,43].

### (e) T cells

T cells orchestrate adaptive immunity, with subsets that either promote pathogen clearance or tolerance. Regulatory T cells (Tregs) are pivotal in limiting excessive inflammation by producing IL-10 and TGF-β and expressing inhibitory molecules like CTLA-4 [44,45]. Tregs balance pro-inflammatory Th1 responses during infections such as tuberculosis and COVID-19 [46], preventing tissue fibrosis and necrosis. Certain Th1-like Tregs restrain CD8+ T cell effector functions to avoid immunopathology [47]. Th2 cells produce IL-10, counteracting Th1-driven inflammation and aiding tolerance. IL-10 broadly regulates immune responses during infections, dampening Th17, CD8+ TIFN-γ cells and other effectors to reduce tissue damage [48,49]. Recent findings suggest that markers of T cell exhaustion arise early in chronic and acute infections, representing an adaptive regulatory state rather than solely a dysfunction from chronic stimulation of TCF1+ precursors [50,51]. Tissue-resident memory T cells maintain local surveillance while minimizing systemic inflammation [52]. The balance between effector and Tregs influences whether the host favours resistance or tolerance, and modulation of these responses could improve disease tolerance.

### (f) Non-immune cells

Non-immune cells—epithelial cells, endothelial cells, fibroblasts and stromal cells—are active participants in disease tolerance. They maintain barriers, regulate immune environments and initiate repair after damage. Epithelial cells produce cytokines and antimicrobial peptides under the influence of IL-17 and IL-22, and they release alarmins (e.g. IL-33) that activate ILCs and other immune cells. They regenerate to maintain barrier integrity and counterbalance oxidative stress, limiting pathogen spread and preserving tissue function [53,54].

Endothelial cells maintain vascular integrity and regulate permeability and leukocyte trafficking. By modulating activation, they can suppress or amplify inflammation. In malaria, a tissue-protective monocyte and macrophage programme is accompanied by an absence of endothelial activation, preserving tissue integrity and limiting organ damage [27]. Liver sinusoidal endothelial cells (LSECs) promote Treg differentiation by tethering TGF-β, contributing to immune regulation [55]. LSECs also clear inflammatory molecules, reducing systemic inflammation. Fibroblasts initially induce inflammation to recruit immune cells but later promote tissue repair by secreting extracellular matrix components, guided by IL-10 and TGF-β to avoid fibrosis [56]. During infection, non-immune cells undergo metabolic and transcriptional reprogramming to survive stress [57], control pathogen spread and coordinate immune responses, making them essential for tissue-specific disease tolerance and potentially healthspan [58].

### (g) Apoptotic cells

Apoptotic cells contribute dynamically to disease tolerance by suppressing inflammation, promoting cell survival and aiding tissue repair. Their rapid clearance prevents release of DAMPs, minimizing inflammation and supporting resolution of infection [59]. For example, apoptotic intestinal epithelial cells limit pathogen leakage and inflammatory responses [60]. While generally protective, dysregulated apoptosis can lead to immune suppression, autoimmunity or fibrosis [61]. Recent data show interplay between apoptosis and senescence programmes [62]; suppression of apoptosis in senescent immune cells can protect regulatory populations, balancing inflammation and aiding recovery. Thus, therapeutically modulating apoptosis requires precision to avoid undermining tolerance mechanisms.

### (h) Senescent cells

Senescent cells, which can potentially arise from any cell type in the body, are characterized by stable cell cycle arrest owing to the overexpression of the cell cycle inhibitors p21 and/or p16, occurring in response to various stressors, including DNA damage, oxidative stress and inflammation [63,64]. Traditionally viewed as detrimental owing to their association with ageing and chronic diseases (figure 2), the impact of senescent cells is largely mediated through the senescence-associated secretory phenotype (SASP), a complex mix of cytokines, chemokines, growth factors and proteases [65]. This secretory profile enables senescent cells to profoundly influence the tissue microenvironment. Consequently, reducing the number of senescent cells has proven effective in lowering age-related deterioration and preventing or delaying the onset of certain pathologies [66–69].

**Figure 2.**
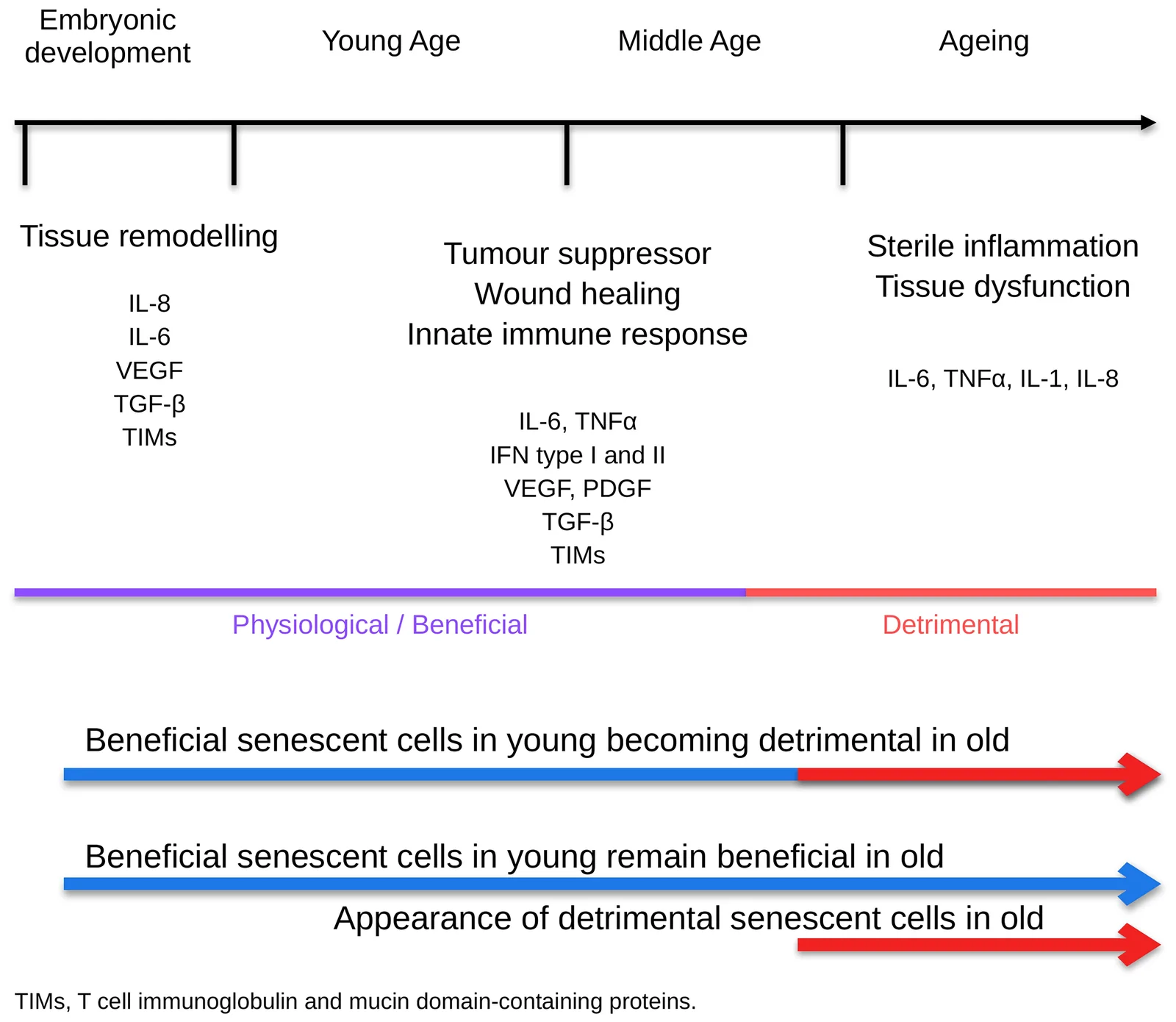
The role of senescence at different stages of life. Activation of senescence can be both beneficial and detrimental. In early life, senescence is often beneficial; however, if senescent cells persist, their prolonged SASP secretion can lead to chronic low-grade inflammation and tissue dysfunction, converting them into a detrimental influence. Short-lived cells, such as certain immune cells, may remain largely beneficial throughout life. By contrast, with ageing, the accumulation of senescent cells among structural and long-lived cell types becomes a source of detrimental effects. Based on this, different therapeutic strategies to improve healthspan could aim either to reverse detrimental senescence back to beneficial states or to selectively ablate detrimental senescent cells while preserving or enhancing beneficial ones. TIMs, T cell immunoglobulin and mucin domain-containing proteins.

Despite these deleterious functions, accumulating evidence supports cellular heterogeneity among senescent cells, with some performing important homeostatic functions during embryonic development and in adult tissues such as skin, liver and lung [70–73] (figure 2). It is currently understood that the beneficial effects of senescent cells depend on their transient nature and timely clearance by immune cells, such as macrophages and NK cells [74,75]. When senescent cells persist—as often occurs in ageing or chronic disease—their prolonged SASP secretion can lead to chronic low-grade inflammation and tissue dysfunction, contributing to conditions such as osteoarthritis, fibrosis and cancer.

Our recent work highlights that p16 activation in the immune compartment plays a strong beneficial role during severe inflammation and tissue damage [76]. One possible explanation for why immune p16^High^ cells exhibit beneficial functions is their relatively rapid turnover, which prevents them from entering a state of prolonged and harmful SASP secretion while allowing for beneficial short-term effects. Using a p16–Cre knock-in mouse model, we observed that all analysed immune cell types accumulate a senescence-like feature, p16 activation, with ageing [76]. Surprisingly, even very short-lived neutrophils exhibited a noticeable fraction of p16^High^ cells. Further analysis showed that, contrary to expectations of a pro-inflammatory SASP, p16^High^ immune cells were primarily anti-inflammatory. For example, p16^High^ tissue-resident peritoneal macrophages (trMacs) expressed known anti-inflammatory and regulatory molecules such as IL-10, Arg1 and Vsig4. Additionally, a notable fraction of p16^High^ T cells were enriched for expression of programmed death-ligand 1 (PD-L1), and the Treg master regulator FoxP3. In trMacs, p16 induction correlated with activation of the ATM–53BP1 DNA damage signalling pathway, rather than accumulation of γH2AX foci, which was mainly associated with p21 induction in a separate trMac subset.

We demonstrated that induction of a p16^High^ programme in immune subsets could be intimately linked to disease tolerance [76]. An increase in p16^High^ cells leading to activation of disease tolerance could be achieved in response to mRNA vaccines, such as the BNT162b2 mRNA COVID-19 vaccine. Mechanistically, we identified TLR7 and stimulator of interferon genes (STING) as potent inducers of p16^High^ immune subsets *in vivo*. We further found that this mRNA vaccine-driven mechanism of p16^High^ immune cell-dependent tolerance had strong protective effects against severe viral, bacterial and sterile inflammation and tissue damage. Indeed, we found that the presence of p16^High^ immune subsets was indispensable for animal survival in response to multiple lethal conditions such as bacterial and LPS-induced sepsis, lethal early COVID-19 infections (90% mice survival after induction of p16^High^ immune subsets compared with 30% in the control group) and lethal ionizing irradiation (40% mice survival after induction of p16^High^ immune subsets compared with 0% in the control group) [76,77]. Importantly, we showed that in humans, a single standard dose of the BNT162b2 mRNA COVID-19 vaccine is sufficient to induce p16 in various immune cells. From a clinical standpoint, we found that p16-expressing immune subsets were significantly reduced in the blood of severe COVID-19 patients, further supporting their critical role in preventing lethal inflammation and severe tissue damage. The clinical efficacy of the BNT162b2 vaccine in reducing hospitalization risk was demonstrated as early as 10 days after the first dose, at a time when the specific adaptive immune response (i.e. antibodies and T cells) has not yet fully developed. Our results could explain these early benefits of vaccination by suggesting a temporary buildup of p16^High^ immune cell-dependent disease tolerance, which limits progression to an inflammatory form of COVID-19, thereby reducing the risk of severe disease and hospitalization. Furthermore, this previously unacknowledged first line of defence in response to mRNA vaccines—by building disease tolerance—could play a vital role in shielding against deadly pathogens while providing sufficient time for the development of long-lasting antibody-based protection.

Based on our results, there are several ways in which p16^High^ immune cells can contribute to disease tolerance. Since p16 induction is tied to strong cell cycle arrest, and senescence programmes often involve induction of anti-apoptotic factors like Bcl-2, these cells may become more resistant to elimination during inflammation. In turn, p16^High^ immune cells are enriched in anti-inflammatory and regulatory-like populations, and thus their improved survival would contribute positively to curbing excessive inflammation. However, if immunosuppression via induction of an anti-inflammatory SASP fails and inflammation continues to escalate, another anti-inflammatory mechanism is triggered. Dying cells, including immune cells, release ATP, which is rapidly converted into adenosine. Adenosine is generally viewed as a suppressor of inflammation, for example, by inhibiting T cell activation and promoting M2 macrophage polarization [78]; however, excessive adenosine accumulation—particularly in ageing or chronic inflammation—can disrupt immune homeostasis. For example, high adenosine levels in aged tissues correlate with chronic inflammation and tissue damage [79,80], and its immunosuppressive effects can contribute to fibrosis or immune exhaustion [81]. In this context, our recent data [77] suggest that p16^High^ immune cells, which are primarily found in immunosuppressive and regulatory subsets, maintain adenosine, in part via upregulation of nicotinamide N-methyltransferase (NNMT) [76,82], at levels that could support their protective functions (e.g. Treg and lymphocyte chemotaxis/migration (LCM) activity) while preventing the deleterious consequences of adenosine overload. In turn, protecting anti-inflammatory and regulatory subsets through activation of the p16^High^ senescence programme could be an important step in preventing immune tolerance in the presence of high levels of adenosine. Furthermore, once inflammation and tissue damage subside, certain immune cells—such as ILCs and tissue-resident macrophages—play a key role in efficient tissue repair. In turn, severe inflammation significantly depletes these cells, while induction of a p16^High^ senescent programme in some of them could protect them and thus enable more effective tissue repair following damage.

Beyond limiting inflammation, p16^High^ immune cells may support tissue homeostasis by protecting stem cell niches [83]. By modulating local cytokine environments and regulating clearance of damaged cells, they could preserve stem cell function and regenerative capacity. However, sustained activation of disease tolerance may potentially involve some trade-offs, including stem cell dysfunction, altered differentiation or even increased cancer risk. Thus, the long-term impact of p16^High^ immune cells probably depends on a balance between tissue protection and maintenance of stem cell fitness within tissue stem cell niches.

Our findings further raise the possibility that building disease tolerance by maintaining increased numbers of p16^High^ immune cell subsets from an early age—before any tissue damage or inflammation—could extend healthspan. This is supported by our observations in *Melanoma Differentiation-Associated gene 5* (*MDA5*)-deficient mice [76]. Similar to the mRNA-based approach, deletion of *MDA5* results in low-to-mild STING activation and induction of p16^High^ immune cell subsets from a young age, translating into improved healthspan. It is tempting to speculate that this mechanism of precautious activation of the p16^High^ state in immune cells, as observed in MDA5-deficient mice, is evolutionarily conserved and active in long-lived animals such as bats and the naked mole rat, supporting their significantly improved healthspan and increased lifespan.

Altogether, our results provide strong evidence for the direct and early benefits of p16^High^ immune cells in promoting disease tolerance and counteracting severe viral, bacterial and sterile inflammation. In turn, sustainable activation of a p16^High^ programme in defined immune subsets from early age—potentially achieved through TLR7 and/or tonic STING activation by inhibiting MDA5—could offer a novel strategy for extending healthspan. Understanding these dynamics opens therapeutic opportunities, such as harnessing senescence's protective roles or selectively clearing detrimental chronic senescent cells to restore tissue health and promote longevity (figure 2).

## 6. Ageing and disease tolerance

Ageing can generally be described as a decline in health over time, measured by both healthspan (how long an individual remains free of age-related disease) and lifespan (the maximum number of years an individual can live). However, there are notable exceptions in several species, such as certain turtles, lobsters, bats, and the naked mole rat, where the probability of death (the chance that an individual of a certain age will die before reaching a later age) remains consistently low. This is in sharp contrast to humans, where the probability of death increases sharply with age. In such species, the low probability of death translates into an extremely low ageing rate, meaning the likelihood of dying does not increase with age. This supports the idea that these animals do not undergo ageing-related tissue and organ decline and therefore do not develop typical age-associated diseases. Understanding how this occurs, and whether similar mechanisms can be introduced in humans, could mark the beginning of the end for diseases like cancer and neurodegeneration.

Ageing is a complex, multifactorial process that, nevertheless, has the potential to be modified or improved. The idea that both healthspan and lifespan can be experimentally extended originates from two seminal studies. In 1917, Osborne *et al.* [84] demonstrated that caloric restriction extends lifespan in rats. Later, researchers discovered that mutating a single gene, *abnormal DAuer Formation-2*, in the worm *Caenorhabditis elegans* could double its lifespan, confirming that genetics also plays a role in ageing [85].

In recent years, there has been significant progress in efforts to better understand and influence ageing. Yet, regardless of the strategy used, improvements appear to have reached a plateau. In mice, for example, gains in healthspan and lifespan typically range between 15 and 30%. Two strategies currently stand out in the effort to combat ageing: caloric restriction and reduction of chronic inflammation. For example, one of the most recent approaches targets the pro-inflammatory molecule IL-11, which led to an increase of approximately 24% in the lifespan of wild-type mice [86]. Other studies have suggested that age-related inflammation can be reduced and healthspan improved by eliminating p16 cells [66], removing senescent cells using a senolytic combination of dasatinib and quercetin [87], or using a senomorphic drug such as fisetin [88]. However, follow-up studies using alternative genetic models or similar drug regimens have not reached similar conclusions [70,89,90]. Therefore, the idea that broad removal of senescent cells is a reliable strategy for improving healthspan or lifespan remains controversial. An additional unresolved question is when any anti-ageing intervention should be applied to achieve the greatest benefit, and whether it is conceivable that there is a point of no return, beyond which very little can be done to reverse—or even delay—ageing-induced tissue deterioration. To address some of these challenges, our recent work proposes a strategy to extend healthspan by promoting disease tolerance from an early age [76]. Rather than attempting broad removal or suppression of senescent cells, we suggest that activating a p16^High^ state in certain immune cells early in life may help to preserve fully active disease tolerance mechanisms. This, in turn, could slow tissue deterioration during ageing, improve healthspan and potentially extend lifespan.

### (a) Changes in disease tolerance during ageing

Ageing affects multiple biological processes, and the gradual changes in disease tolerance is one of them (figure 1). These changes occur for numerous reasons, making it challenging to fully re-establish disease tolerance in older individuals. A continuous buildup of tissue damage with age, combined with a simultaneous reduction in the number and activity of tissue remodelling cell types and mechanisms, invariably leads to the development of a more pro-inflammatory environment, which further exacerbates age-related decline. The ability of disease tolerance mechanisms to counteract excessive inflammation—for example, by activating survival pathways in p16^High^ cells such as PD-L1+ T cells, Tregs or IL-10-producing macrophages—appears to peak in middle-aged mice [77]. Beyond that point, additional immunosuppression to counterbalance ever-increasing tissue damage and inflammation with ageing may depend on less specific mechanisms, such as the release of ATP from dying cells, followed by its conversion into immunosuppressive adenosine. However, adenosine is a potent inhibitor of disease tolerance, and its levels must be tightly controlled before any effective restoration of disease tolerance can occur [77].

Another factor contributing to the age-related changes in disease tolerance is the decrease in the number of reparative cell types or a shift in their properties. For example, aged alveolar type 2 (AT2) cells exhibit telomere shortening, reduced surfactant production and impaired differentiation into AT1 cells. This impairs alveolar repair after viral infection and increases susceptibility to acute respiratory distress syndrome. Single-cell analyses show that senescent AT2 cells upregulate MHC class I expression, suggesting aberrant immune activation that may contribute to autoinflammatory lung injury [91]. Concurrently, mitochondrial ROS accumulation in bronchial epithelial cells activates the NLRP3 inflammasome, driving IL-1β production and neutrophil-driven inflammation. This mechanism underlies the progression of chronic obstructive pulmonary disease, in which oxidative stress overwhelms antioxidant defences like glutathione [92].

Another hallmark of ageing that contributes to disease tolerance is the change of tissue-resident macrophages towards pro-inflammatory states. This shift has been well documented in multiple tissues, including the heart, liver and adipose tissue. These macrophages secrete pro-inflammatory cytokines such as TNF-α and IL-6, establishing a chronic, low-grade inflammatory environment [93]. In adipose tissue, this inflammation disrupts adipokine signalling, reduces adiponectin levels, impairs glucose homeostasis and amplifies oxidative stress [94]. This environment reduces disease tolerance by weakening antioxidant defences and increasing susceptibility to infections or metabolic complications. Moreover, studies have shown that embryo-derived tissue-resident macrophage populations decline with age in tissues such as the liver and skin. This loss in tissue remodelling activity is not fully compensated by recruitment of haematopoietic stem cell (HSC)-derived monocytes, leading to a deficit in maintaining tissue homeostasis [95,96]. Ageing is also associated with a reduction in ILCs, further impairing tissue repair after inflammation or injury and contributing to changes in disease tolerance with age [97].

Taken together, these findings suggest that multiple factors contribute to changes in disease tolerance mechanisms with ageing and, in turn, its restoration in old organisms could be a complex and difficult task. On the other hand, activating and maintaining disease tolerance mechanisms from an early age, as we recently proposed in studies using MDA5 knockout mice [76], could be an interesting alternative strategy to improve healthspan.

### (b) Building disease tolerance as a healthspan-extending strategy from a young age

Bats are emerging as a fascinating model for studying ageing and disease tolerance owing to their negligible ageing rate, which translates into exceptional longevity, coupled with unique immune adaptations and the ability to tolerate viral infections without succumbing to disease. Bats are well known to be natural reservoirs of viruses, including coronaviruses (SARS-CoV and MERS-CoV) and filoviruses (Ebola and Marburg), among others. By contrast to humans and other mammals that experience chronic low-grade inflammation with age (inflammaging), bats regulate inflammation differently, which may contribute to both their longevity and their ability to tolerate infections without excessive tissue damage. Instead of eliminating pathogens entirely (resistance), bats often tolerate infections by controlling the immune response to limit tissue damage (disease tolerance), for example, by upregulating antiviral pathways without triggering excessive inflammation [98].

Among other potential mechanisms, the capping of excessive inflammation in bats could be linked to reduced DNA-sensing capabilities. For example, the PYHIN family of genes, which are involved in DNA sensing, is absent [99], while the STING pathway is less functional in bats [100]. The STING protein in bats lacks a critical serine residue, which results in dampened STING activation and reduced IFN responses to DNA viruses; this, in turn, may help to prevent excessive inflammation [3]. Similarly, lowering STING in aged mice improves muscle strength, endurance and cognitive functions, suggesting that STING's role in ageing is more nuanced than previously thought [101]. These findings suggest that manipulating pathways that control interferon and inflammatory responses could be key to maintaining disease tolerance and enhancing lifespan. It is important to emphasize that, in order to achieve beneficial effects, it is critical to cap such pathways at a certain level rather than to fully inactivate them. Full inactivation of such pathways could have strong negative consequences, as seen in cGAS knockout mice, which display an accelerated ageing phenotype characterized by increased frailty, inflammation and shortened lifespan in females [102]. Similarly, full knockout of STING, instead of partial lowering STING in aged mice [101], leads to a shortening of lifespan along with increased pro-inflammatory cytokines, indicating that basal STING signalling may be important to sustain tissue function and to support organismal longevity [103].

Our recent data further support the notion that maintaining so-called ‘tonic’ STING activation from an early age could have a strong positive impact on healthspan and lifespan in mice. We found that the depletion of MDA5 maintains low STING activation that is sufficient to induce p16 expression in defined immune subsets and significantly increases disease tolerance from a young age [77]. MDA5, encoded by the *IFIH1* gene, is a crucial cytoplasmic sensor for viral double-stranded RNAs in the innate immune system and a member of the RIG-I-like receptor family. It plays a vital role in detecting viral infections and initiating antiviral responses. Upon detecting viral dsRNA, MDA5 activates the type I interferon signalling pathway, leading to the production of interferons and inflammatory cytokines [104,105]. Unexpectedly, we found that knockout of MDA5 results in modest activation of the STING pathway. The mechanism is not clear; however, one possible explanation is that while both MDA5 and STING can sense RNA–DNA duplexes of roughly similar size, the absence of RNA sensing via MDA5 may result in compensatory upregulation of the STING pathway. Further analysis of MDA5 knockout mice revealed that they appear visibly less aged, with a significantly reduced frailty index compared with their wild-type littermates. The expression of genes related to inflammation in aged wild-type and MDA5 knockout animals—particularly IL-6, IL-1α and TNF-α—was downregulated, while IL-10, a cytokine related to immune regulation, was overexpressed. Furthermore, MDA5 knockout animals preserved HSC function during ageing by maintaining metabolic fitness and proteostasis [83]. These data show that it is possible to delay tissue deterioration by manipulating specific pathways associated with disease tolerance.

The study of disease tolerance and the role of beneficial senescence via induction of a p16^High^ state specifically in immune subsets offers a promising avenue for treating certain conditions and potentially improving healthspan and lifespan. By manipulating specific pathways, including those that lead to the activation of p16^High^ immune cells, such as following the inactivation of MDA5, it may be possible to maintain enhanced disease tolerance from a young age, thereby significantly delaying ageing-induced tissue deterioration. These findings highlight the potential for therapeutic strategies aimed at enhancing disease tolerance as a means to combat age-related diseases, offering an interesting strategy for improving human health and longevity.

## Data Availability

This article has no additional data.

## Theme

One contribution of 21 to a theme issue ‘Developmental circuits in ageing’.
